# Supplementary material for: Modeling the Phase Equilibria of Associating Polymers in Porous Media with Respect to Chromatographic Applications
Source: Polymers (Basel). 2022 Aug 4;14(15):3182. doi: 10.3390/polym14153182 (PMC9370872; doi:10.3390/polym14153182)
Supplement: Supplementary file 1 [file polymers-14-03182-s001.zip › polymers-1826431-supplementary.pdf]

Supplementary Material

Modeling the Phase Equilibria of Associating Polymers in Porous Media with Respect to Chromatographic Applications

Xiu Wang [a], Zuzana Limpouchová [b], Karel Procházka [b*], Rahul Kumar Raya [b], and Yonggang Min [a*]

[a] *School of Materials and Energy, Guangdong University of Technology, Guangzhou 510006, Guangdong, China*

[b] *Department of Physical and Macromolecular Chemistry, Faculty of Science, Charles University, Hlavova 8, 128 43, Prague 2, Czech Republic*

Corresponding authors:

Karel Procházka: karel.prochazka@natur.cuni.cz

Yonggang Min: ygmin@gdut.edu.cn

Table of Contents

The bulk-pore model S3

The modification of CBMC algorithm S4

Additional results S9

Simulations starting at different initial conditions S9

Comparison between the bulk-pore partitioning and the bulk solution S11

The number-average association numbers S12

Distributions of association numbers S13

References S16

The bulk-pore model

The employed bulk-pore model for simulation of copolymer phase equilibria including the partitioning between two phases, is illustrated in Figure S1. The unconfined cubic box with the dimensions of 100 × 100 × 100 ($L_{x,bulk} \times L_{y,bulk} \times L_{z,bulk}$) represents the bulk mobile phase and the impermeable cylindrical tube with a variable diameter, D, models the porous stationary phase. For a given pore diameter, D, we change $L_{x,pore}$ to keep the volume ratio V_{pore} / V_{bulk} = 1. For both the bulk and the pore, periodic boundary conditions (PBC) are imposed in all directions except the impermeable shell of the cylindrical pore.

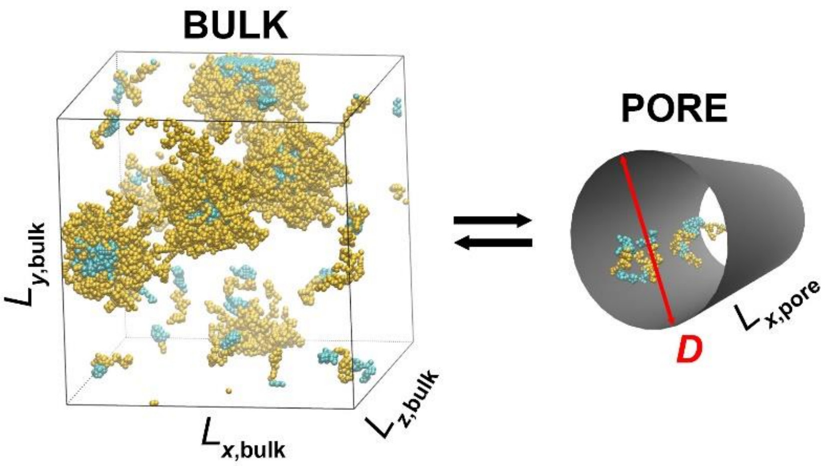

Figure S1. Schematic representation of the employed bulk-pore model.

The modification of CBMC algorithm

The used modified configurational-bias Monte Carlo (CBMC) algorithm [1, 2] stems from the original CBMC method invented by Siepmann and Frenkel [3] and explicitly takes in account the non-zero interactions between polymers and solvent beads on a cubic lattice.

The original CBMC method uses the so-called implicit solvent. It means that each unoccupied lattice position is supposed to contain a solvent bead. Simultaneously, all interaction parameters with the solvent are zero, i.e., $\varepsilon_{\alpha S} = 0$, where S stands for the solvent and α can be both the polymer segments (A and B for the studied diblock copolymers) and the solvent bead, and the solvent quality is modeled indirectly by the nonzero interactions between polymer beads, $\varepsilon_{\alpha\beta}$ (α, $\beta \neq S$). A randomly selected copolymer chain is deleted from the box, i.e., the lattice sites originally occupied by monomers (A or B) are replaced by the solvent beads (S), and a new conformation of this chain is subsequently generated. The old chain is labeled as *x* and the new one with the newly grown conformation is denoted as *y*.

During the reconstruction of a chain, the energy of a trial position, *j*, for the subsequent segment of the chain under reconstruction is evaluated in each step, *i*. The selection probability, P_i, for the segment location *i* ($i > 1$) is given by the formula [3]

$$P_i = \frac{\exp(-u_i/k_\mathrm{b}T)}{\sum_{j=1}^{q} \exp\left[-\left(u_j\right)_i/k_\mathrm{b}T\right]} = \frac{\exp(-u_i/k_\mathrm{b}T)}{\omega_i}, \tag{1}$$

where u_i represents the energy of segment *i* at the actual position and *q* is the number of all possible positions for this segment. The summation runs over all free positions labeled as *j* around the ($i-1$)-th segment and $\left(u_j\right)_i = \sum \varepsilon_{\alpha\beta}$ is the energy of *i*-th segment on lattice site *j*, i.e., the summation over all neighbor positions around the lattice site *j*, where the energy interaction parameter, $\varepsilon_{\alpha\beta}$, describes the pairwise energy

of neighbors α and β. The Rosenbluth weight for a given state, i.e., a given polymer conformation, is defined as the product of weights of individual segments,

$$W_N = \prod_{i=1}^{N} \omega_i, \tag{2}$$

where N represents the total chain length. The probability of generation of copolymer in the state y, p_y, is the product of probabilities of generation of individual segments,

$$p_y = \prod_{i=1}^{N} \frac{\exp(-u_{i,y}/k_\mathrm{b}T)}{\omega_{i,y}}, \tag{3}$$

where the subscript "i,y" stands for segment i in state y. Obviously, the probability, p_y, equals

$$p_y = \frac{\exp[-(\sum_{i=1}^{N} u_{i,y})/k_\mathrm{b}T]}{W_{N,y}} = \frac{\exp(-U_y/k_\mathrm{b}T)}{W_{N,y}}, \tag{4}$$

where

$$U_y = \sum_{i=1}^{N} u_{i,y} = \sum_{i=1}^{N} \left(\sum \varepsilon_{\alpha\beta}\right)_{i,y} \tag{5}$$

and accordingly

$$p_x = \frac{\exp[-(\sum_{i=1}^{N} u_{i,x})/k_\mathrm{b}T]}{W_{N,x}} = \frac{\exp(-U_x/k_\mathrm{b}T)}{W_{N,x}}, \tag{6}$$

where

$$U_x = \sum_{i=1}^{N} u_{i,x} = \sum_{i=1}^{N} \left(\sum \varepsilon_{\alpha\beta}\right)_{i,x}. \tag{7}$$

Here U_y and U_x stand for the energy of the entire chain of length N at state y and state x, respectively. Equations 4 and 6 hold only if all pairwise interactions with solvent are zero, i.e., $\varepsilon_{\alpha\mathrm{S}} = 0$.

The detailed balance condition requires that the probability of generating state x from state y is the same as that from y to x, i.e.,

$$\pi_x p_y P_{\mathrm{acc},y} = \pi_y p_x P_{\mathrm{acc},x}, \tag{8}$$

where $\pi_x = \exp(-U_x/k_\mathrm{b}T)$ is the probability of state x, $\pi_y = \exp(-U_y/k_\mathrm{b}T)$ is the probability of state y, and $P_{\mathrm{acc},y}$ (or $P_{\mathrm{acc},x}$) is the probability that the new state y (or x) is

accepted. If we replace the p_y and p_x by Equations 4 and 6, respectively, Equation 8 can be written as

$$\exp\left(\frac{-U_x}{k_b T}\right)\frac{\exp(-U_y/k_\mathrm{b}T)}{W_{N,y}}P_{\mathrm{acc},y} = \exp\left(\frac{-U_y}{k_b T}\right)\frac{\exp(-U_x/k_\mathrm{b}T)}{W_{N,x}}P_{\mathrm{acc},x} \quad (9)$$

and consequently,

$$\frac{P_{\mathrm{acc},y}}{W_{N,y}} = \frac{P_{\mathrm{acc},x}}{W_{N,x}}. \quad (10)$$

Hence one of possible choices of the acceptance probability of the new trial conformation, i.e., state y, which fulfills the detailed balance condition, is

$$P_{\mathrm{acc},y} = \min\left(1, \frac{W_{N,y}}{W_{N,x}}\right), \quad (11)$$

and the acceptance probability of the old conformation, i.e., state x, is $1-P_{\mathrm{acc},y}$ [3]. This simple acceptance criterion derived by Siepmann and Frenkel stems from the fact that the probability of generating state y, p_y, can be expressed as the ratio of the Boltzmann factor of generated state, $\exp(-U_y/k_\mathrm{b}T)$, to the Rosenbluth weight of the state, $W_{N,y}$, as shown in Equation 4. Note that this method can be used only for the systems in which the interaction parameters of solvent with any other segments are zero, i.e., $\varepsilon_{\alpha\mathrm{S}} = 0$.

The original CBMC algorithm cannot be directly applied for studying copolymers in selective solvents, because the interactions of polymer segments with solvent molecules are non-zero and both the polymer segments and solvent molecules, the positions of which changed during the reconstruction of the chain, contribute to ΔU of the whole system. To elucidate the necessity of the CBMC modification, below we schematically depict a possible reconstruction of a short self-avoiding walk (SAW) chain on a simple 2D lattice. It is obvious that the energy difference is $\Delta U = 2(\varepsilon_\mathrm{BA} - \varepsilon_\mathrm{BS})$. In other words, ΔU contains the contribution of polymer segment-solvent interactions.

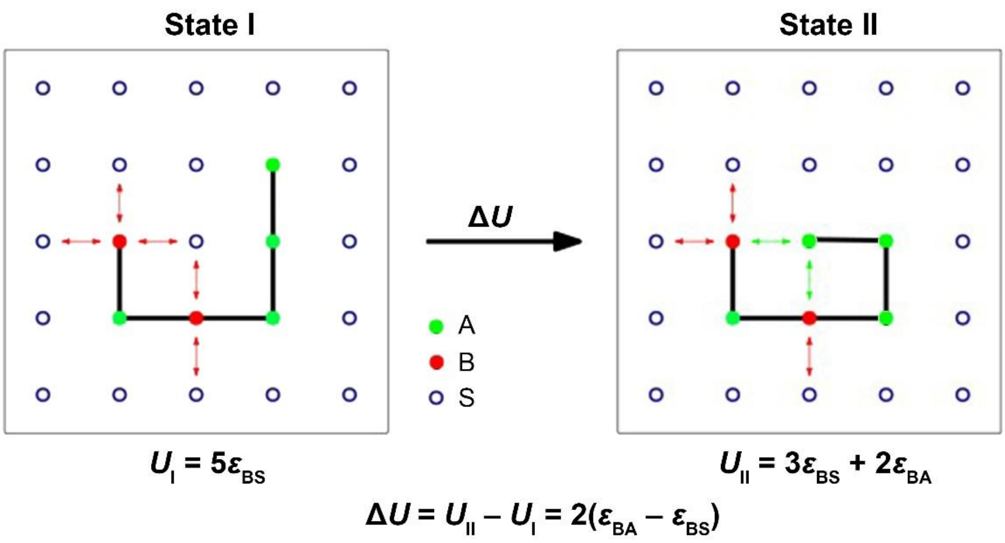

Figure S2. The simplest illustrative example on a 2D lattice. The empty blue circles represent solvent beads (S), and the green and red disks represent A and B beads, respectively. The thick black lines represent the SAW chain and the red arrows show lattice neighbor B and S beads, and the green ones show the B and A neighbor pairs. The only non-zero interaction parameters are ε_{BS} and ε_{BA} ($\varepsilon_{BS} \neq \varepsilon_{BA}$), and all others pairwise interactions are zero.

Technically, the modification of CBMC method consists in two changes. First, the energy difference parameter, $z_{\alpha\beta} = \varepsilon_{\alpha\beta} - \varepsilon_{S\beta}$, where S denotes the solvent and α, β can be A, B or W (pore wall), is defined and the total energy difference of a monomer α at given lattice position, Z, is calculated as the sum of all $z_{\alpha\beta}$ of its lattice neighbors, i.e., $Z = \sum z_{\alpha\beta}$. Second, the energy change of the whole simulation box instead of the energy of a chain under reconstruction is taken in account.

The detailed balance condition for the modified algorithm is analogous to that in the original CBMC (Equation 8), however with $z_{\alpha\beta}$ instead of $\varepsilon_{\alpha\beta}$. Note that the states y and x differ in N=n_A+n_B positions of n_A segments A and n_B segments B**.** Then the total energy in the states x and y can be expressed as

$$U_{\text{box}}^{x} = U_{\text{box}}^{0} + \sum_{i=1}^{N} Z_i^x , \tag{12}$$

and

$$U_{\text{box}}^{y} = U_{\text{box}}^{0} + \sum_{i=1}^{N} Z_i^y , \tag{13}$$

respectively, where U^0_{BOX} is the partial energy of the simulation box containing the solvent and the part of the copolymer system, which does not change and is common for both states *y* and *x*. Therefore, U^0_{BOX} can be expressed as

$$U^0_{\mathrm{box}} = U^x_{\mathrm{box}} - \sum_{i=1}^N Z_i^x = U^y_{\mathrm{box}} - \sum_{i=1}^N Z_i^y \,. \tag{14}$$

After the substitution in the detailed balance condition, we obtain

$$\exp\left(\frac{-U^x_{\mathrm{box}}}{k_{\mathrm{b}}T}\right)\prod_{i=1}^N\left[\frac{\exp(-Z_i^y/k_{\mathrm{b}}T)}{\omega_{i,y}}\right]P_{\mathrm{acc},y} = \exp\left(\frac{-U^y_{\mathrm{box}}}{k_{\mathrm{b}}T}\right)\prod_{i=1}^N\left[\frac{\exp(-Z_i^x/k_{\mathrm{b}}T)}{\omega_{i,x}}\right]P_{\mathrm{acc},x}, \tag{15}$$

and subsequently

$$\exp\left[\frac{-(U^x_{\mathrm{box}}-\sum_{i=1}^N Z_i^x)}{k_{\mathrm{b}}T}\right]\frac{P_{\mathrm{acc},y}}{W_{N,y}} = \exp\left[\frac{-(U^y_{\mathrm{box}}-\sum_{i=1}^N Z_i^y)}{k_{\mathrm{b}}T}\right]\frac{P_{\mathrm{acc},x}}{W_{N,x}}. \tag{16}$$

So formally the identical relation,

$$\frac{P_{\mathrm{acc},y}}{W_{N,y}} = \frac{P_{\mathrm{acc},x}}{W_{N,x}}, \tag{17}$$

is obtained and the same acceptance probability of the new trial conformation (state *y*),

$$P_{\mathrm{acc},y} = \min\left(1, \frac{W_{N,y}}{W_{N,x}}\right), \tag{18}$$

can be used.

In this simulation study, the values of all interaction parameters are expressed in the energy units, $k_{\mathrm{b}}T_0$, where T_0 represents the temperature of reference state. The majority of the simulations was performed at the temperature $T/T_0 = 1.7$ and we also varied the temperature to investigate its effects.

Additional results

Simulations starting at different initial conditions

We performed two sets of simulations starting from the bulk and the pore, i.e., all chains were initially placed either in the bulk or in the pore. The curves of partition coefficient, K, vs. total concentration, C, are shown in Figure S3. The data obtained from two sets of simulations for the systems with the pores of which the sizes, D, range from 15 to 30 are almost identical, which confirms the anticipated equilibrium and ergodicity.

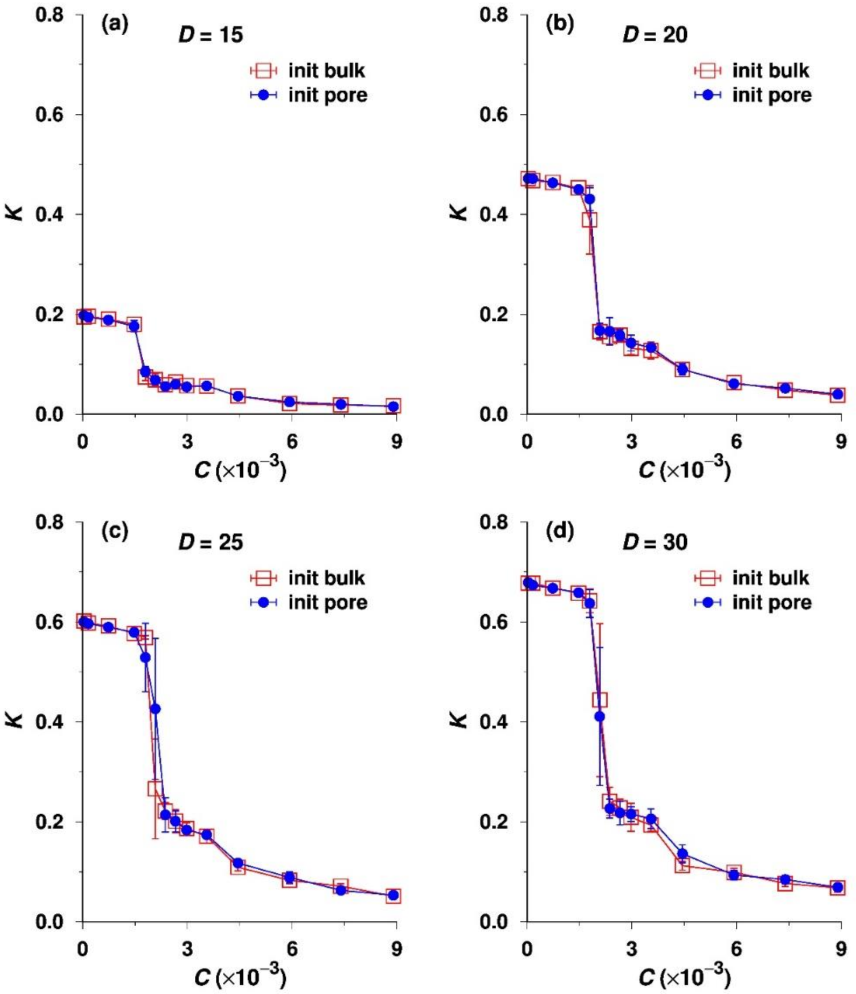

Figure S3. Partition coefficient K, as a function of the total concentration, C, for the A32B32 diblock copolymers partitioning between the bulk and the pore for different diameters, D. The legends "init bulk" and "init pore" represent the data obtained from the two sets of simulations starting from the bulk and the pore, i.e., all chains were initially in the bulk and in the pore, respectively. The interaction strengths between two beads are $\epsilon_{SS} = \epsilon_{AA} = \epsilon_{BB} = 0$, $\epsilon_{AS} = 0$, $\epsilon_{BS} = 0.1$ and $\epsilon_{AB} = 0.15$. The cylindrical pore is inert, i.e., $\epsilon_{WS} = \epsilon_{WA} = \epsilon_{WB} = 0$. We set the temperature $T/T_0 = 1.7$, where T_0 represents the temperature of reference state.

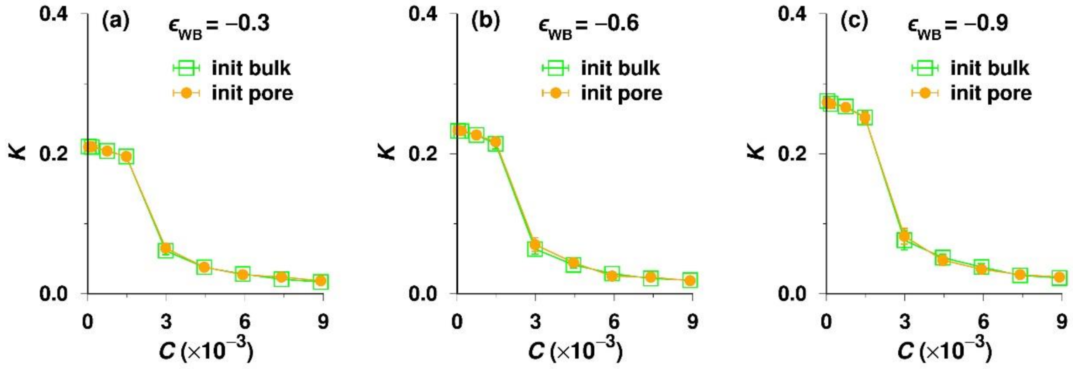

Figure S4. Partition coefficient, K, as a function of total concentration, C, for the A32B32 diblock copolymers partitioning between the bulk and the narrow cylindrical pore of diameter D = 15. The pore wall is inert for block A and the solvent, i.e., $\epsilon_{WA} = \epsilon_{WS} = 0$, but attractive for block B, i.e., $\epsilon_{WB} < 0$. Panels a to c represent different ϵ_{WB}. The legends "init bulk" and "init pore" represent the data obtained from the two sets of simulations starting from the bulk and the pore, i.e., all chains were initially in the bulk and in the pore, respectively. The interaction strengths between two beads are $\epsilon_{SS} = \epsilon_{AA} = \epsilon_{BB} = 0$, $\epsilon_{AS} = 0$, $\epsilon_{BS} = 0.1$ and $\epsilon_{AB} = 0.15$. We set the temperature $T/T_0 = 1.7$.

Nevertheless, the results of two sets of simulations for the wide pore of D = 60 differ for both SEC (presented and discussed in the main text) and IC conditions (see Figure S5). While the simulations starting with all chains in the bulk obviously generate the equilibrium data, the K values for the simulations starting with the considerably concentrated solutions of chains in the pore are unrealistically large, indicating the freezing of simulations in arrested states upon the formation of crowded and stacked micellar solutions in the pore at C > CMC.

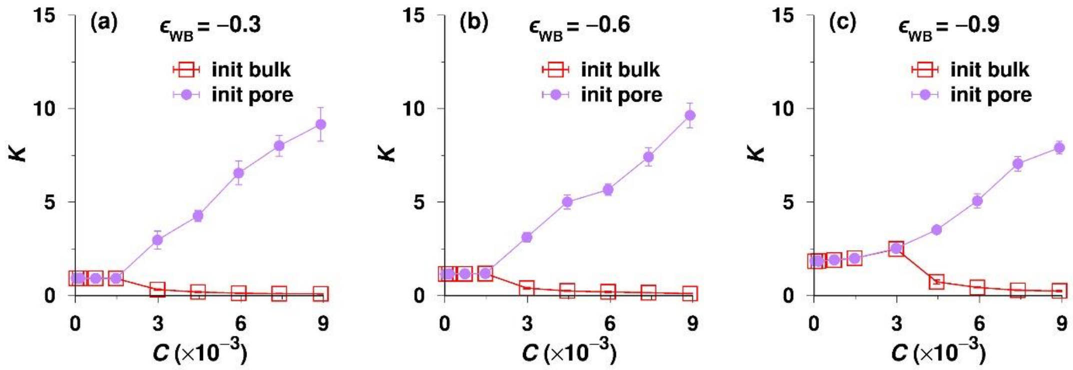

Figure S5. Partition coefficient, K, as a function of total concentration, C, for the A32B32 diblock copolymers partitioning between the bulk and the wide cylindrical pore of diameter D = 60. The pore wall is inert for block A and the solvent, i.e., $\epsilon_{WA} = \epsilon_{WS} = 0$, but attractive for block B, i.e., $\epsilon_{WB} < 0$. Panels a to c represent distinct ϵ_{WB}. The legends "init bulk" and "init pore" represent the data obtained from the two sets of simulations starting from the bulk and the pore, i.e., all chains were initially in the bulk and in the pore, respectively. The interaction strengths between two beads are $\epsilon_{SS} = \epsilon_{AA} = \epsilon_{BB} = 0$, $\epsilon_{AS} = 0$, $\epsilon_{BS} = 0.1$ and $\epsilon_{AB} = 0.15$. We set the temperature $T/T_0 = 1.7$.

Comparison between the bulk-pore partitioning and the bulk solution

We plot the number of non-associated free A32B32 chains, n_{free}, in the bulk phase of the bulk-pore partitioning against C_{bulk} in Figure S6a, where the concentration of beads in the bulk phase, C_{bulk}, is calculated as

$$C_{\mathrm{bulk}} = \frac{C(V_{\mathrm{eff,pore}}+V_{\mathrm{eff,bulk}})}{KV_{\mathrm{eff,pore}}+V_{\mathrm{eff,bulk}}}. \quad (19)$$

Here C is the total concentration of beads in the entire bulk-pore system, K is the partition coefficient, and $V_{\mathrm{eff,pore}}$ and $V_{\mathrm{eff,bulk}}$ represent the effective volumes of the pore and the bulk, respectively. The n_{free} vs. C_{bulk} curve obtained from the bulk solutions only is also included in Figure S6a, and in this case $C_{\mathrm{bulk}}=nN/V_{\mathrm{bulk}}$, where n denotes the total number of copolymer chains. Moreover, we plot the partition coefficient, K, against C_{bulk}, in Figure S6b. The curves presented in Figure S6a and b indicate that the CMCs provided by the bulk-pore partitioning simulations are consistent with that computed from the bulk solutions.

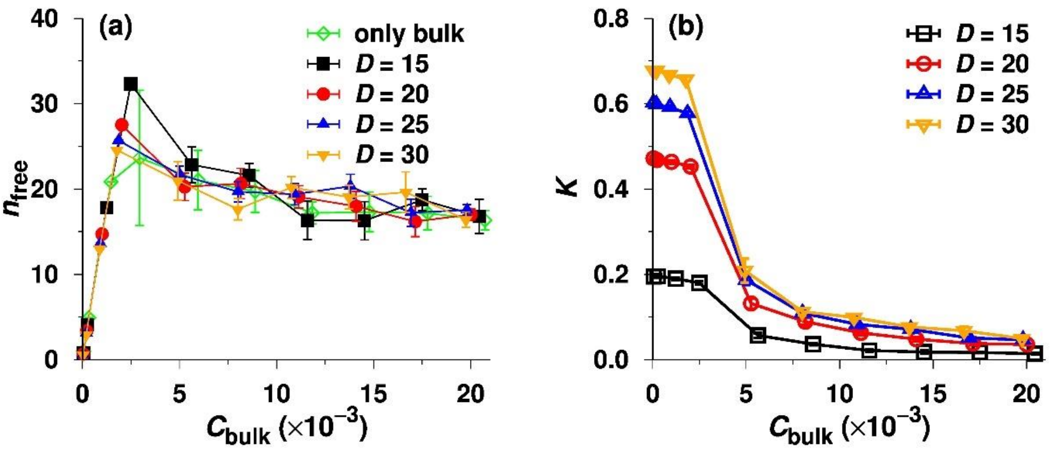

Figure S6. (a) Number of free A32B32 chains, n_{free}, in the bulk as a function of the concentration of beads in the bulk phase of the bulk-pore partitioning, C_{bulk}, for various pore diameters, D. There is an exception of simulations performed only in the bulk solutions (see the green empty diamonds), i.e., without the bulk-pore partitioning. (b) Partition coefficient, K, as a function of C_{bulk}, for various D. The interaction strengths between two beads are $\epsilon_{\mathrm{SS}} = \epsilon_{\mathrm{AA}} = \epsilon_{\mathrm{BB}} = 0$, $\epsilon_{\mathrm{AS}} = 0$, $\epsilon_{\mathrm{BS}} = 0.1$ and $\epsilon_{\mathrm{AB}} = 0.15$. The cylindrical pore is inert, i.e., $\epsilon_{\mathrm{WS}} = \epsilon_{\mathrm{WA}} = \epsilon_{\mathrm{WB}} = 0$. We set the temperature $T/T_0 = 1.7$.

The number-average association numbers

From the simulations of partitioning of A32B32 diblock copolymers between the bulk and pore, we evaluated the number-average association numbers,

$$\overline{A_s} = \frac{\sum_{A_s} A_s n(A_s)}{\sum_{A_s} n(A_s)}, \tag{20}$$

in the bulk phase. Here $n(A_s)$ stands for the simulated number of associates formed by A_s copolymer chains. We plot $\overline{A_s}$ against the concentration of beads in the bulk, C_{bulk} (see Equation 19), for various pore diameters, D, in Figure S7. The data also include those from the simulations individually performed only in the bulk, i.e., without bulk-pore partitioning. In the fairly dilute regime, $\overline{A_s}$ is quite low and constant, i.e., only slightly higher than zero. With increasing C_{bulk}, it rapidly increases to the maximum and then plateaus. This observed trend is consistent with the closed association scheme [4] and with the previously discussed concentration dependence of partition coefficient, K.

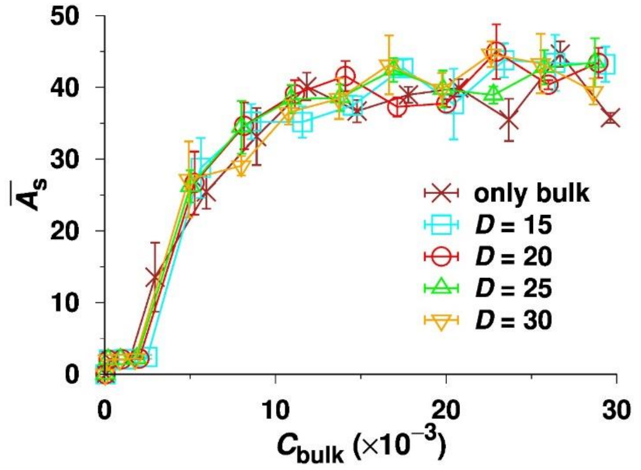

Figure S7. Number-average association number, $\overline{A_s}$, of A32B32 diblock copolymers in the bulk phase, which partition between the bulk and the pore, as a function of the concentration of beads in the bulk, C_{bulk}, for various pore diameters, D. There is an exception of simulations performed only in the bulk solutions (see the brown crosses), i.e., without the bulk-pore partitioning. The interaction strengths between two beads are $\epsilon_{SS} = \epsilon_{AA} = \epsilon_{BB} = 0$, $\epsilon_{AS} = 0$, $\epsilon_{BS} = 0.1$ and $\epsilon_{AB} = 0.15$. The cylindrical pore is inert, i.e., $\epsilon_{WS} = \epsilon_{WA} = \epsilon_{WB} = 0$. We set the temperature $T/T_0 = 1.7$.

Distributions of association numbers

We also assessed the number distribution of association numbers,

$$m_{\mathrm{n}}(A_{\mathrm{s}}) = \frac{n(A_{\mathrm{s}})}{\sum_{A_{\mathrm{s}}} n(A_{\mathrm{s}})}, \tag{21}$$

and the weight distribution of association numbers,

$$m_{\mathrm{w}}(A_{\mathrm{s}}) = \frac{A_{\mathrm{s}} n(A_{\mathrm{s}})}{\sum_{A_{\mathrm{s}}} A_{\mathrm{s}} n(A_{\mathrm{s}})}, \tag{22}$$

of associates, where $n(A_{\mathrm{s}})$ stands for the number of associates consisting of A_{s} copolymer chains. We plot $m_{\mathrm{n}}(A_{\mathrm{s}})$ and $m_{\mathrm{w}}(A_{\mathrm{s}})$ for various pore diameters, D, including the simulations performed only in bulk, and various bulk concentrations, C_{bulk}, in Figures S8 and S9, respectively.

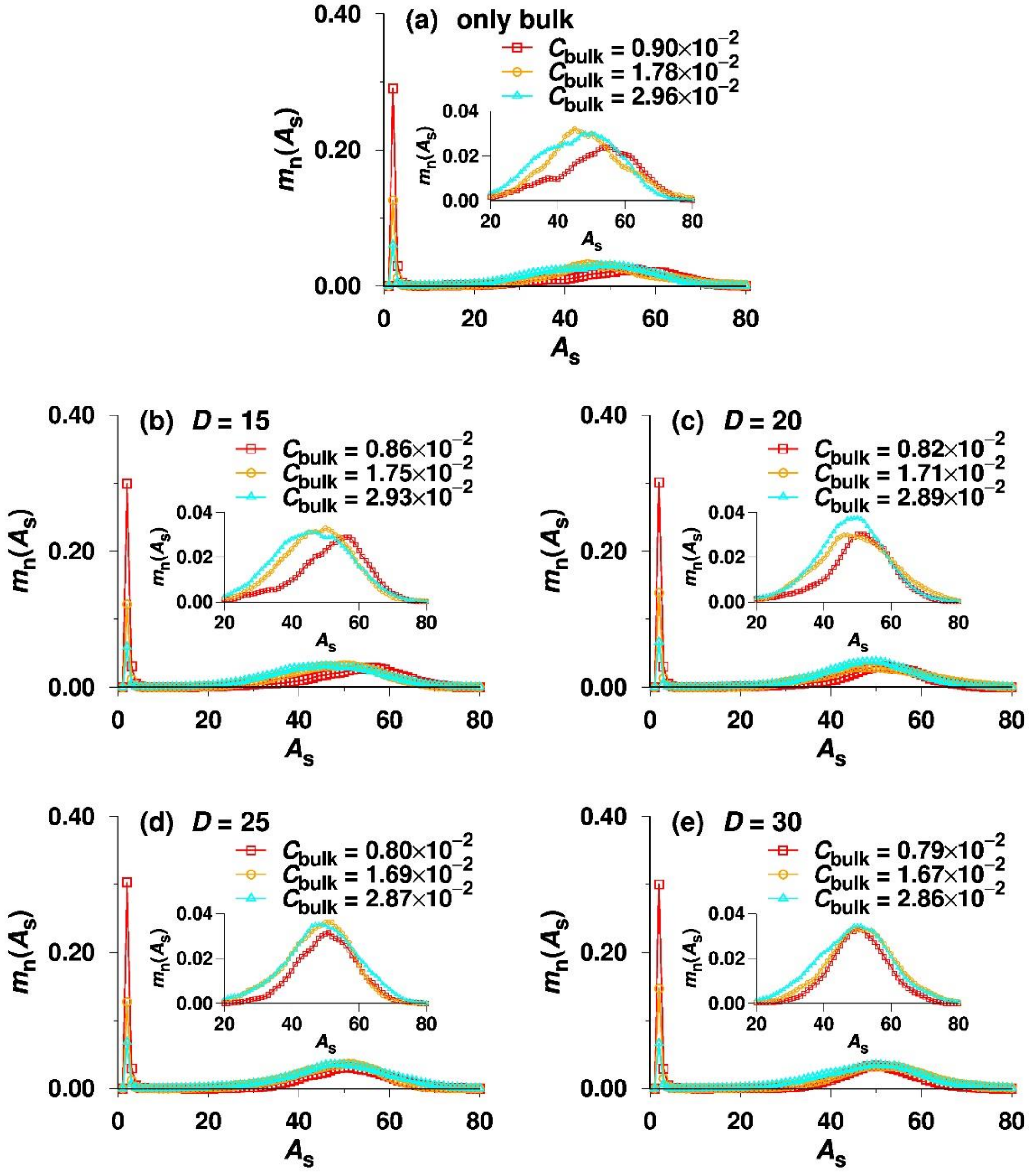

Figure S8. Plots of the number distributions of associates, $m_n(A_s)$, for the A32B32 diblock copolymers in the bulk phase. Panel a represents the data from the simulations performed only in the bulk and panels b to e represent the partitioning with various pore diameters, D. We zoom in the curves ranging from 20 to 80 (A_s) in the insets. The interaction strengths between two beads are $\epsilon_{SS} = \epsilon_{AA} = \epsilon_{BB} = 0$, $\epsilon_{AS} = 0$, $\epsilon_{BS} = 0.1$ and $\epsilon_{AB} = 0.15$. The cylindrical pore is inert, i.e., $\epsilon_{WS} = \epsilon_{WA} = \epsilon_{WB} = 0$. We set the temperature $T/T_0 = 1.7$.

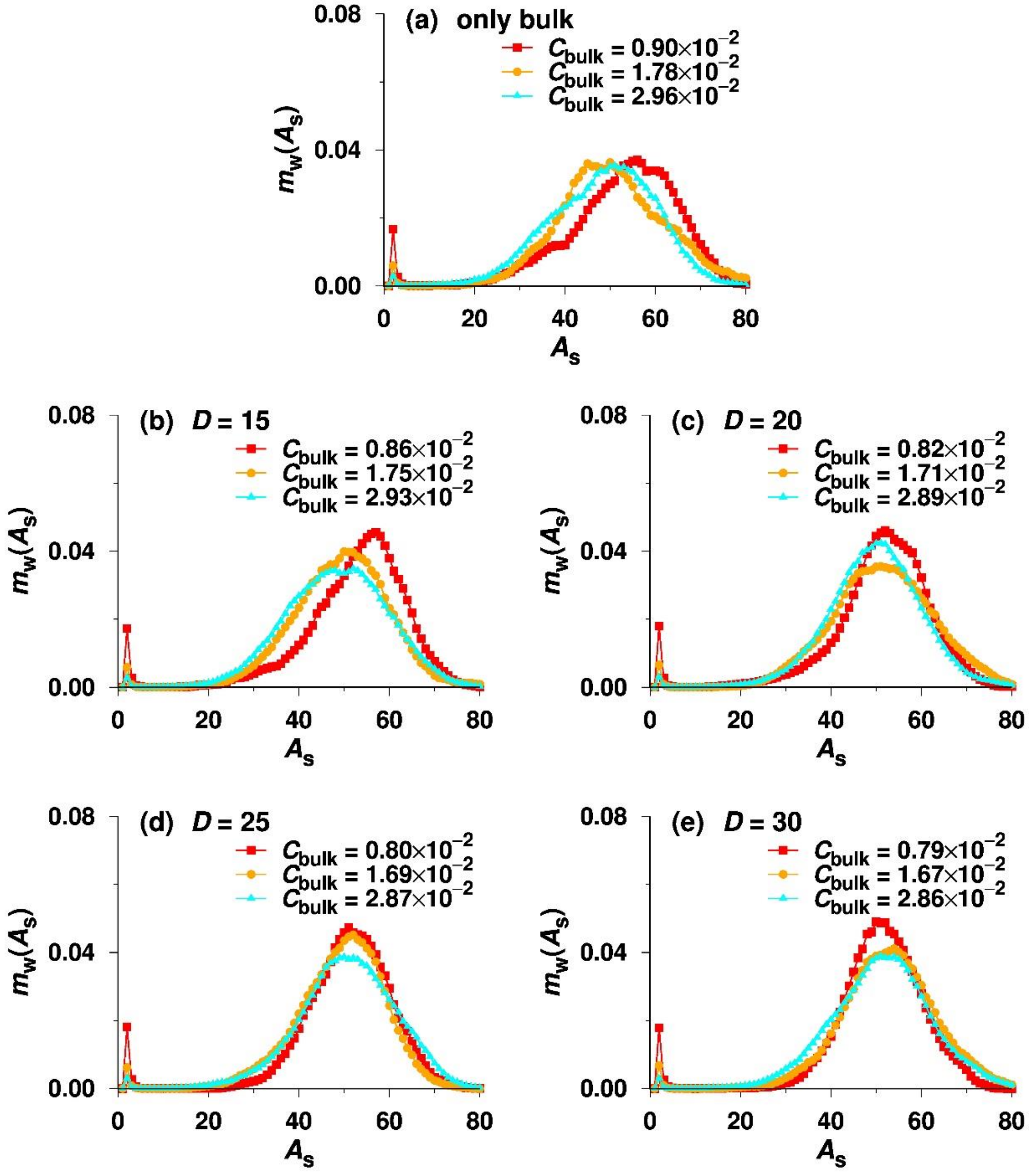

Figure S9. Plots of the weight distributions of associates, $m_w(A_s)$, for the A32B32 diblock copolymers in the bulk phase. Panel a represents the data from the simulations performed only in the bulk and panels b to e represent the partitioning with various pore diameters, D. The interaction strengths between two beads are $\epsilon_{SS} = \epsilon_{AA} = \epsilon_{BB} = 0$, $\epsilon_{AS} = 0$, $\epsilon_{BS} = 0.1$ and $\epsilon_{AB} = 0.15$. The cylindrical pore is inert, i.e., $\epsilon_{WS} = \epsilon_{WA} = \epsilon_{WB} = 0$. We set the temperature $T/T_0 = 1.7$.

References

1. Havránková, J.; Limpouchová, Z.; Procházka, K. A new simulation algorithm with revised "association criteria" for studying the association of heteroarm star copolymers. *Macromolecular Theory and Simulations* **2005**, 14, 560–568.
2. Kuldová, J.; Košovan, P.; Limpouchová, Z.; Procházka, K. Computer study of the association behavior of gradient copolymers: analysis of simulation results based on a new algorithm for recognition and classification of aggregates. *Macromolecular Theory and Simulations* **2013**, 22, 61–70.
3. Siepmann, J.I.; Frenkel, D. Configurational bias Monte Carlo: a new sampling scheme for flexible chains. *Molecular Physics* **1992** , 75, 59–70.
4. Elias, H.G.; Šolc, K. Multimerization: Association and aggregation, 14. Distinction between open and closed associations. *Die Makromolekulare Chemie* **1975**, 176, 365–371.
